# Polypharmacy, hospitalization, and mortality risk: a nationwide cohort study

**DOI:** 10.1038/s41598-020-75888-8

**Published:** 2020-11-03

**Authors:** Tae Ik Chang, Haeyong Park, Dong Wook Kim, Eun Kyung Jeon, Connie M. Rhee, Kamyar Kalantar-Zadeh, Ea Wha Kang, Shin-Wook Kang, Seung Hyeok Han

**Affiliations:** 1 Department of Internal Medicine, National Health Insurance Service Medical Center, Ilsan Hospital, Goyangshi, Gyeonggi-do Republic of Korea; 2 Research and Analysis Team, National Health Insurance Service Medical Center, Ilsan Hospital, Goyangshi, Gyeonggi-do Republic of Korea; 3 Department of Big Data, National Health Insurance Service, Wonju-si, Gangwon-do Republic of Korea; 4 Department of Pharmacy, National Health Insurance Service Medical Center, Ilsan Hospital, Goyangshi, Gyeonggi-do Republic of Korea; 5 Harold Simmons Center for Kidney Disease Research and Epidemiology, University of California Irvine School of Medicine, Orange, CA USA; 6 Nephrology Section, Tibor Rubin Veterans Affairs Medical Center, Long Beach, CA USA; 7 Department of Internal Medicine, Yonsei University College of Medicine, 50-1 Yonsei-ro, Seodaemun-Gu, Seoul, 03722 Republic of Korea

**Keywords:** Health care, Medical research, Risk factors

## Abstract

Polypharmacy is a growing and major public health issue, particularly in the geriatric population. This study aimed to examine the association between polypharmacy and the risk of hospitalization and mortality. We included 3,007,620 elderly individuals aged ≥ 65 years who had at least one routinely-prescribed medication but had no prior hospitalization within a year. The primary exposures of interest were number of daily prescribed medications (1–2, 3–4, 5–6, 7–8, 9–10, and ≥ 11) and presence of polypharmacy (≥ 5 prescription drugs per day). The corresponding comparators were the lowest number of medications (1–2) and absence of polypharmacy. The study outcomes were hospitalization and all-cause death. The median age of participants was 72 years and 39.5% were men. Approximately, 46.6% of participants experienced polypharmacy. Over a median follow-up of 5.0 years, 2,028,062 (67.4%) hospitalizations and 459,076 (15.3%) all-cause deaths were observed. An incrementally higher number of daily prescribed medications was found to be associated with increasingly higher risk for hospitalization and mortality. These associations were consistent across subgroups of age, sex, residential area, and comorbidities. Furthermore, polypharmacy was associated with greater risk of hospitalization and death: adjusted HRs (95% CIs) were 1.18 (1.18–1.19) and 1.25 (1.24–1.25) in the overall and 1.16 (1.16–1.17) and 1.25 (1.24–1.25) in the matched cohorts, respectively. Hence, polypharmacy was associated with a higher risk of hospitalization and all-cause death among elderly individuals.

## Introduction

Polypharmacy refers to the prescription of multiple medications to a single individual, and it can be defined either numerically or qualitatively^1–5^. Some studies have described polypharmacy as the use of a higher number of drugs than clinically indicated^2^. However, this definition is based on a clinical judgement that is difficult to operationalize in large studies^5^. By contrast, the numeric definition can vary according to practice setting or research protocol, and it is often used in most epidemiologic studies due to its simplicity (e.g., ≥ 5 prescription medications)^3–5^. Although there is no universal definition of polypharmacy to date, it is considered a growing and major public health problem worldwide, particularly among the geriatric population in whom there is a high burden chronic comorbidities^1,2,5^.

Some patients with multiple comorbidities require the simultaneous prescription of pharmaceutical products. However, a growing body of evidence suggests that drugs are oftentimes prescribed inappropriately. Moreover, polypharmacy may be associated with adverse outcomes particularly in the elderly population^1,5^. In fact, several symptoms related to the central nervous system and gastrointestinal tract are caused by adverse drug events, and additional drugs are often prescribed to control these symptoms^5–7^. Such multiple drug combinations may lead to drug-drug interactions and heighten the risk of adverse effects^5–7^. Furthermore, the use of multiple drugs can increase pill burden and medical costs^3^. Concurrently, the prescription of multiple medications may negatively affect patient adherence and health-related quality of life (HRQOL)^8^. Older individuals are particularly vulnerable to unexpected drug-related problems because of the multiple drug regimens, higher number of comorbid conditions, and age-associated physiological changes in pharmacokinetics and pharmacodynamics with respect to certain drugs^5,9^.

There have been a number of studies that examined the relationship between polypharmacy and adverse events. The prevalence of polypharmacy has consistently increased, which is particularly evident among elderly individuals^9–12^. Predictably, the number of prescribed medications increases concomitantly with age and number of comorbidities^13^. To date, several epidemiologic studies reported possible detrimental associations of polypharmacy with falls^14^, renal failure^15^, frailty^16^, poor physical function^17^, and cognitive impairment^18,19^. In addition, patients consuming multiple medications are more likely to experience poor HRQOL^20,21^ and frequent hospitalizations^22–24^. More importantly, in a recent meta-analysis that pooled data across 47 studies, polypharmacy, defined as ≥ 5 prescribed drugs, was associated with a 31% higher risk for mortality^25^. However, not all studies have confirmed this association^26,27^. Nevertheless, interpretation of these aforementioned studies are limited by potential residual confounders and confounding by indication (i.e., higher number of medications prescribed to those with ill health), medication ascertainment by self-report (i.e., which results in exposure misclassification), and examination of drug exposure within a short period (i.e., ~ 30 days) (i.e., which limits the examination of long-term exposure-outcome associations). Thus, to better inform the field, we sought to examine the association of polypharmacy with the risk of hospitalization and death in a large longitudinal cohort of elderly community-indwelling individuals from the Korean National Health Insurance Service (NHIS) database linked to the nationwide pharmacy claims data.

## Results

### Baseline characteristics of the study population

The flow chart of study cohort construction and the baseline characteristics of 3,007,620 participants who met the eligibility criteria of the study are shown in Supplementary Fig. S1 and Table 1, respectively. The median (inter-quartile range, IQR) age of the participants was 72 years (68–77 years), among whom 39.5% (95% confidence interval [CI], 39.4%-39.6%) were men, 86.3% (95% CI, 86.3%-86.3%) were urban residents, and 81.5% (95% CI, 81.5%-81.5%) had at least one comorbidity based on the Charlson comorbidity index (CCI). In the study population, the mean (standard deviation, SD) and median (IQR) numbers of daily prescribed medications were 4.9 (3.2) and 4.0 (2.0–7.0), respectively. Individuals who were prescribed with a greater number of medications were more likely to be older and have a higher prevalence of comorbidities. Overall, the distributions of sex and residential area were similar across the groups.

**Table 1:** Baseline characteristics of 3,007,620 participants stratified by the number of daily prescribed medications.

Characteristic	Overall	Number of routinely-prescribed medications (per day)					
		1 to 2	3 to 4	5 to 6	7 to 8	9 to 10	≥ 11
Number of participants	3,007,620	764,673	841,498	618,087	388,520	211,000	183,842
Number of medications	4.9 ± 3.2	1.6 ± 0.5	3.5 ± 0.5	5.5 ± 0.5	7.4 ± 0.5	9.4 ± 0.5	12.9 ± 2.4
**Age, years**	73.4 ± 6.3	72.6 ± 6.2	73.1 ± 6.3	73.7 ± 6.3	74.0 ± 6.3	74.3 ± 6.2	74.3 ± 6.0
65–69 years, %	31.5	37.2	33.3	29.5	26.7	24.9	23.8
70–74 years, %	31.0	30.8	31.2	31.1	30.9	30.6	31.1
75–79 years, %	20.7	17.9	19.7	21.5	23.0	24.2	25.3
≥ 80 years, %	16.9	14.1	15.8	18.0	19.5	20.4	19.8
Gender, % men	39.5	38.5	40.1	39.8	39.2	39.3	40.6
**Residential area, %**							
Large city	43.5	44.6	43.8	43.1	42.5	42.4	42.5
Small city	42.8	42.1	42.8	43.0	43.2	43.1	43.8
Rural area	13.7	13.2	13.4	13.9	14.3	14.4	13.7
**CCI scores**	2.0 ± 1.7	1.3 ± 1.4	1.7 ± 1.5	2.2 ± 1.7	2.6 ± 1.8	3.0 ± 2.0	3.6 ± 2.0
0, %	18.5	32.4	22.4	12.5	7.5	4.7	2.5
1, %	27.6	32.1	31.8	28.0	22.4	17.6	11.4
2, %	22.2	19.1	22.3	24.9	24.9	23.1	18.9
≥ 3, %	31.7	16.5	23.5	34.7	45.2	54.6	67.3
**Comorbid conditions, %**							
Hypertension	79.5	65.0	79.1	84.1	88.6	91.1	93.2
Diabetes	36.9	19.9	29.8	42.2	51.5	58.5	67.1
Acute myocardial infarction	1.9	0.4	1.0	2.4	3.4	4.2	5.0
Heart failure	4.7	1.4	3.1	5.7	7.7	9.4	11.1
Cerebrovascular disease	13.9	5.0	10.1	16.5	21.1	24.9	30.6
Hemiplegia	1.0	0.3	0.6	1.2	1.6	2.0	2.5
Dementia	6.8	4.0	5.2	7.2	9.1	10.9	14.0
Peripheral vascular disease	20.4	13.3	20.1	22.4	24.3	23.0	29.5
Liver disease	5.9	5.2	5.6	6.1	6.4	6.8	7.7
Chronic pulmonary disease	26.3	23.2	24.1	26.4	29.0	31.6	36.3
Connective tissue disease	6.3	4.8	5.3	6.4	7.6	8.8	10.9
Peptic ulcer disease	34.7	31.0	31.9	35.2	38.0	41.0	46.5
Chronic kidney disease	1.3	0.5	0.8	1.3	2.0	2.8	4.2
Malignancy	6.7	6.9	6.2	6.5	6.7	7.1	8.0

Data are presented as means ± standard deviation, number, or percentages.

*CCI* Charlson comorbidity index.

### Relationship between the number of medications and risk of hospitalization or death

During a median follow-up period of 5.0 years, 2,028,062 (67.4%; 95% CI, 67.3%-67.5%) hospitalizations and 459,076 (15.3%; 95% CI, 15.3%-15.3%) deaths from any cause were observed, with corresponding crude incidence rates of 228.5 (95% CI, 228.2–228.8) and 32.5 (95% CI, 32.4–32.5) per 1000 person-years, respectively. An incrementally higher number of daily prescribed medications was associated with a stepwise increase in outcome event rates (Supplementary Fig. S2).

**Figure 1:**
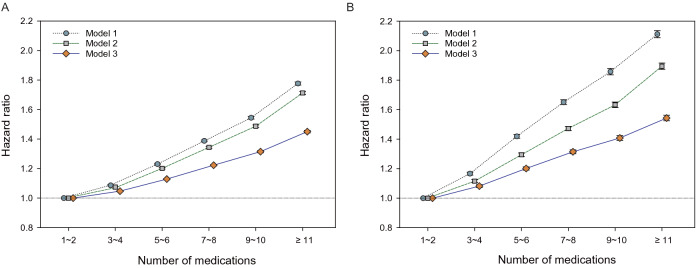
Associations between the number of daily prescribed medications with the risk of (**A**) hospitalization and (**B**) mortality. All models were adjusted for age, sex, residential area, and Charlson comorbidity index score.

**Figure 2:**
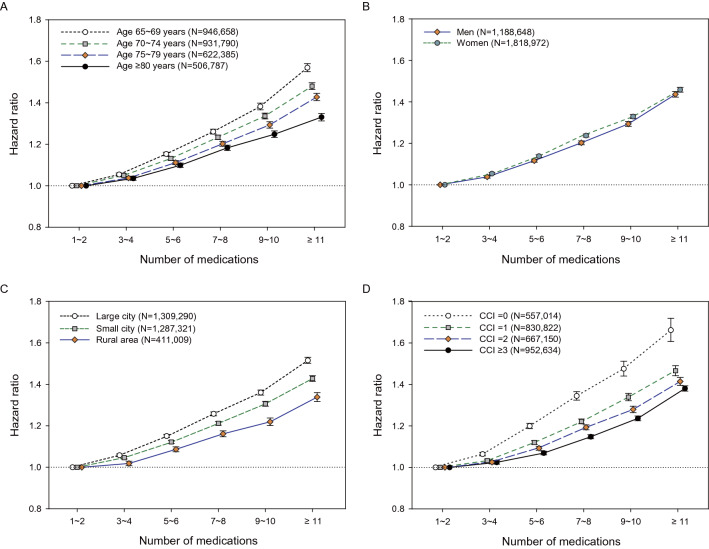
Subgroup analyses of the association between the number of daily prescribed medications and the risk of hospitalization. Subgroup analyses were performed and stratified by (**A**) age (65–69, 70–74, 75–80, and ≥ 80 years), (**B**) sex (men and women), (**C**) residential area (large city, small city, and rural area), and (**D**) comorbidity burden (CCI scores: 0, 1, 2, and ≥ 3). All models were adjusted for age, sex, residential area, and CCI score. *CCI* Charlson comorbidity index.

**Figure 3:**
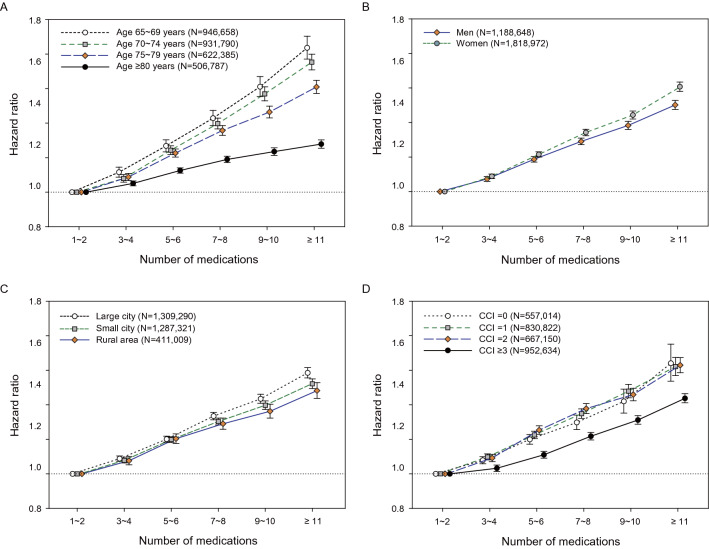
Subgroup analyses of the association between the number of daily prescribed medications and the risk of all-cause mortality. Subgroup analyses were performed and stratified by (**A**) age (65–69, 70–74, 75–80, and ≥ 80 years), (**B**) sex (men and women), (**C**) residential area (large city, small city, and rural area), and (**D**) comorbidity burden (CCI scores: 0, 1, 2, and ≥ 3). All models were adjusted for age, sex, residential area, and CCI score. *CCI* Charlson comorbidity index.

In the Cox regression models adjusted for socio-demographic data and comorbidities, there was a graded association between the number of daily prescribed medications and the risks of hospitalization and death (Fig. 1 and Table 2). Considering a potential heterogeneous population, we conducted subgroup analyses stratified by age, sex, residential area, and comorbidity burden. We found that the graded relationships between the number of medications and adverse outcomes were consistent across these subgroups (Figs. 2 and 3 and Supplementary Table S1).

**Table 2:** Associations between the number of daily prescribed medications and adverse outcomes.

Number of medications	Crude event rate (per 1000 PYs)		Model 1			Model 2			Model 3		
	Incidence	95% CI	HR	95% CI	*p*	HR	95% CI	*p*	HR	95% CI	*p*
**Hospitalization**											
1 to 2	191.6	(191.1–192.2)	1.00			1.00			1.00		
3 to 4	208.4	(207.8–208.9)	1.09	(1.08–1.09)	< 0.001	1.07	(1.07–1.08)	< 0.001	1.05	(1.04–1.05)	< 0.001
5 to 6	236.4	(235.7–237.1)	1.23	(1.22–1.24)	< 0.001	1.20	(1.20–1.21)	< 0.001	1.13	(1.12–1.13)	< 0.001
7 to 8	267.3	(266.3–268.3)	1.39	(1.38–1.39)	< 0.001	1.34	(1.34–1.35)	< 0.001	1.22	(1.22–1.23)	< 0.001
8 to 10	298.1	(296.6–299.6)	1.54	(1.54–1.55)	< 0.001	1.49	(1.48–1.50)	< 0.001	1.31	(1.31–1.32)	< 0.001
≥ 11	344.1	(342.3–345.8)	1.78	(1.77–1.79)	< 0.001	1.71	(1.70–1.72)	< 0.001	1.45	(1.44–1.46)	< 0.001
**Death**											
1 to 2	23.3	(23.2–23.5)	1.00			1.00			1.00		
3 to 4	27.7	(27.5–27.8)	1.17	(1.16–1.18)	< 0.001	1.12	(1.11–1.13)	< 0.001	1.08	(1.07–1.09)	< 0.001
5 to 6	34.4	(34.2–34.6)	1.42	(1.41–1.43)	< 0.001	1.29	(1.28–1.31)	< 0.001	1.20	(1.19–1.21)	< 0.001
7 to 8	40.8	(40.5–41.1)	1.65	(1.63–1.67)	< 0.001	1.47	(1.46–1.49)	< 0.001	1.31	(1.30–1.33)	< 0.001
8 to 10	47.0	(46.5–47.4)	1.86	(1.84–1.88)	< 0.001	1.63	(1.61–1.65)	< 0.001	1.41	(1.39–1.42)	< 0.001
≥ 11	54.1	(53.6–54.6)	2.11	(2.09–2.14)	< 0.001	1.89	(1.87–1.92)	< 0.001	1.54	(1.52–1.56)	< 0.001

Adjustments in model (1): unadjusted; model (2): age, sex, residential area; and model (3): all covariates in model (2) plus Charlson comorbidity index score.

*PYs* person-years, *HR* hazard ratio, *CI* confidence interval.

### Polypharmacy and risk of hospitalization or death

We then sought to examine the association between polypharmacy and the risk of hospitalization and death. The overall prevalence rate of polypharmacy was 46.6% (95% CI 46.5–46.7%). In the overall cohort, individuals with polypharmacy were older and had a higher comorbidity burden compared to those without polypharmacy (Supplementary Table S2). In the multivariate Cox models adjusted for age, sex, residential area, and CCI score, polypharmacy was found to be associated with significantly a higher risk of hospitalization and mortality (adjusted HRs [95% CIs]: 1.18 (1.18–1.19) and 1.25 (1.24–1.25), respectively).

In the propensity score analyses, we identified 1,070,337 matched pairs of individuals with vs. without polypharmacy whose baseline characteristics were well balanced (Supplementary Table S2). In the matched cohort, there were 1,463,971 (68.4%; 95% CI, 68.3–68.5%) hospitalizations and 332,782 (15.6%; 95% CI, 15.6–15.6%) mortality cases. Similar to the findings observed in the overall cohort, polypharmacy was associated with a significantly higher risk of hospitalization and mortality. The corresponding HRs (95% CI) for each outcome were 1.16 (1.16–1.17) and 1.25 (1.24–1.25), respectively (Supplementary Table S3). Moreover, the Kaplan–Meier analyses revealed that individuals with polypharmacy had a significantly higher rate of hospitalization and mortality than those without polypharmacy in both the overall and matched cohorts (Supplementary Figs. S3 and S4).

## Discussion

In this large national longitudinal cohort study, we found a graded association between the number of medications and the risk of adverse clinical outcomes among individuals aged 65 years or older. This association was consistent across clinically relevant subgroups stratified according to age, sex, residential area, and comorbidity burden. Furthermore, polypharmacy was associated with a significantly higher risk of hospitalization and mortality in both the overall and propensity-score matched cohorts. Thus, polypharmacy might have ill effects on the health and survival of elderly people.

With increasing longevity, individuals are more likely to present with a higher number of health problems as well as treatment with multiple drugs. Accordingly, there has been a growing concern that polypharmacy may result in adverse drug-drug interactions, particularly in the elderly population^5–7^. For example, a previous study using the Swedish Drug Register showed a strong relationship between the number of dispensed drugs and possible drug-drug interactions^28,29^. Although drugs are provided to improve patients’ health status, the risk of prescribing multiple concomitant drugs may outweigh individual benefits due to potential ensuing side effects^6,30^. To date, several studies have shown that the use of multiple medications is associated with a broad range of adverse clinical events including falls, fractures, kidney impairment, frailty, poor HRQOL, cognitive dysfunction, and hospitalization^14–24^. However, in these studies, it is difficult to determine whether poor health status or the prescription of multiple drugs occurred first. For example, a high number of prescribed drugs may be a proxy for illness, without direct causal link with adverse outcomes, making it difficult to estimate the effects polypharmacy on health status. Despite rigorous adjustment for comorbidities, confounding by illness may persist in observational studies examining the association between polypharmacy and health outcomes.

Although polypharmacy has been associated with the abovementioned complications, studies examining the relationship between polypharmacy and mortality risk have been mixed. A meta-analysis by Leelakanok et al.^25^ found a dose–response relationship between the number of prescribed medications and mortality. However, this analysis examined a highly heterogeneous population and could not rule out the possibility of residual confounding affecting the pooled estimates. In contrast, two consecutive studies by Schöttker et al.^31,32^ sought to mitigate the influence of confounding by indication using two approaches. In the analyses that performed multivariate adjustment for chronic conditions, polypharmacy was found to be associated with a two-fold higher risk of non-cancer related mortality in older German adults^31^. However, in analyses that examined cohorts who were matched upon baseline characteristics using propensity scores, the associations were attenuated to the null^31^. Nevertheless, in another observational study conducted in Taiwan in which polypharmacy and comorbidities were examined as time-varying exposures, polypharmacy was not associated with a higher death risk among adults aged 65 years and older^23^.

In the present study, we sought to address the issue of confounding by indication using three approaches, which were as follows: 1) adjustment of comorbidity index, 2) subgroup analysis stratified by CCI score, and 3) propensity-score matching analyses. In the conventional Cox models adjusted for disease burden, a higher number of daily prescribed medications was associated with a greater risk of hospitalization and death. Moreover, graded associations were largely similar across all subgroups stratified by CCI score. Furthermore, in the propensity-score matched analyses, individuals with polypharmacy had a 1.2- and 1.3-fold higher risk of hospitalization and mortality. In summary, all three approaches demonstrated a consistent association between polypharmacy and a higher risk of hospitalization and death. To the best of our knowledge, to date, this is the largest study that examined over three million elderly adults in Korea, thereby providing a strong statistical power. While the underlying mechanisms responsible for polypharmacy-related adverse outcomes should be further investigated, our findings highlight the need to identify strategies that can reduce polypharmacy in clinical practice and motivate more judicious prescription of multiple medications, particularly in the geriatric population.

In relation to this, there have been efforts to reduce polypharmacy and inappropriate prescribing practices through educational interventions or alert systems^33–36^. One of these studies included the D-PRESCRIBE (Developing Pharmacist-Led Research to Educate and Sensitize Community Residents to the Inappropriate Prescriptions Burden in the Elderly), which is a recent randomized controlled trial involving older adults in Quebec^36^. This study showed that a pharmacist-led educational intervention resulted in greater discontinuation of inappropriately prescribed medications after six months^36^. However, at present, most studies have only examined the short-term effects of interventions on prescribing patterns. Hence, further long-term studies are required to investigate whether strategies for reducing polypharmacy can significantly improve “hard” clinical endpoints such as hospitalization and mortality.

Several limitations of our study bear mention. First, given that the Korean NHIS database only captures information on prescribed medication, data on the use of over-the-counter, complementary, and alternative drugs were not obtained. In addition, drugs prescribed for short-term intervals were not considered and detailed information on the duration of drug use and the number of medications before study entry was not collected. Furthermore, there was no information on long-acting drugs prescribed once a week or month and other types of medications such as injections, inhalers, eye drops, and topical agents. These drugs might also result in adverse reactions. Hence, the current study might have underestimated the actual prevalence of polypharmacy in this population. Moreover, we determined the number of medications consumed based on prescription data. However, they do not convey information on adherence to the prescribed medications nor the actual number of pills taken by the patients. Second, our definition of polypharmacy was based on a cutoff of ≥ 5 prescriptions, which has been used in prior studies^12,13,19,25,28,31–33^. However, this threshold may not be applicable to all patients. Therefore, future studies are needed to ascertain inappropriate prescriptions using more sophisticated approaches such as the Beers^37^ or the STOPP (Screening Tool of Older Peoples Prescriptions)^38^ criteria. In a recent study by Nam et al.^39^, the prevalence of potentially inappropriate prescribing (PIP) was remarkably high among Korean elderly individuals based on the 2012 Beers criteria. Currently, we are collecting relevant data to analyze the association between PIP and clinical consequences in corollary studies. Third, although a series of sensitivity analyses were conducted to try to account for potential confounding by indication and reverse causation, residual confounding could not be excluded. In fact, the number of individuals without any CCI comorbidity had at least one prescribed medication. Thus, they are likely to have other comorbid conditions that might not be captured by CCI score, thereby causing confounding effects. Finally, this study was conducted on non-institutionalized elderly individuals; thus, our findings might only be generalizable to community-dwelling elderly individuals not to those living in nursing homes or younger populations.

In conclusion, polypharmacy was associated with a higher risk of hospitalization and all-cause mortality in a large national cohort of Korean elderly individuals. These findings underscore the risk of prescribing multiple pharmaceutical products and highlight the urgent need for judicious and appropriate prescription practices and identification of strategies that avoid polypharmacy in the geriatric population.

## Methods

### Source population

We obtained data from the Korean NHIS database, which is linked to nationwide pharmacy claims data. Since NHIS, which is a single-payer national health system, covers compulsory health insurance for all citizens in Korea, all medical records of covered inpatient and outpatient visits are centralized in the NHIS database. These include diagnostic codes, procedures, prescriptions, medical costs, and personal information (e.g., age, sex, residential area, and death records)^40,41^. The pharmacy claims database provides details on all prescription medications for each individual, which include drug names (generic and brand names), start and end dates of prescription, number of days for drug supply, and prescribed doses.

The source population comprised 6,100,982 elderly individuals aged ≥ 65 years who were captured in the 2012 NHIS database. We first excluded 1,876,821 individuals who were hospitalized or who died during the exposure period from January 1, 2012, to December 31, 2012. To determine the association between the number of routinely prescribed medications and adverse outcomes, we further excluded 1,216,541 individuals who were not prescribed any medications or who received a prescription for ≤ 270 days in 2012. Therefore, the final study population comprised 3,007,620 individuals (Supplementary Fig. S1). The study was carried out in accordance with the Declaration of Helsinki. The Institutional Review Board of NHIS Ilsan Hospital approved this study, and the need for informed consent was waived as only deidentified data were examined.

### Data collection and measurements

Baseline data on sociodemographic information such as age, sex, and residential area were collected in the year of study entry. Comorbidities (e.g., hypertension, diabetes, acute myocardial infarction, congestive heart failure, cerebrovascular disease, hemiplegia, dementia, peripheral vascular disease, liver disease, chronic pulmonary disease, connective tissue disease, peptic ulcer disease, chronic kidney disease, and malignancy) were assessed using *the International Statistical Classification of Disease and Related Health Problems, Tenth Revision,* coding algorithms. The presence of comorbidities was confirmed by the presence of at least one diagnostic code identified up to one year prior to the exposure period. Moreover, the CCI score was calculated as a proxy of disease burden and illness severity^42^.

Data on outpatient prescription medications were identified from the pharmacy claims database and were extracted using specific drug-class codes or names. To determine the risk associated with longer periods of drug exposure, we only selected “routinely-prescribed” medications, which was defined as medications prescribed for ≥ 270 days from January 1, 2012, to December 31, 2012. The number of drugs was counted according to the fifth level of the Anatomical Therapeutic Chemical (ATC) Classification System^43^. Drugs which had the same fifth level of ATC code were considered as one drug. In the case of combined products containing two or more active components, the number of drugs was calculated as the sum of the number of each active ingredient. Only drugs of oral formulations were included, and other types of drugs such as injections, inhalers, eye drops, and topical agents were excluded. Moreover, long-acting drugs prescribed once a week or month were excluded.

### Exposure and outcome ascertainment

The co-primary exposures of interest were the (1) number of daily prescribed medications and (2) presence vs. absence of polypharmacy. The number of daily prescribed drugs was categorized into six groups (1–2, 3–4, 5–6, 7–8, 9–10, and ≥ 11), and polypharmacy was defined as the use of ≥ 5 prescription drugs per day.

The study outcomes of interest were hospitalization and all-cause mortality, which were assessed via a time-to-first-event analysis. Follow-up started on January 1, 2013 (index date) and ended during the occurrence of any event or until December 31, 2017 (study end date), which ever came first.

### Statistical analysis

Data from the descriptive analyses were summarized using means (SD), medians (IQR), or proportions according to data type. To determine potential dose–response relationships, we first examined the associations between the number of medications and study outcomes using the Cox proportional hazard regression models with three incremental levels of adjustment. These levels were as follows: (1) Model 1: unadjusted; (2) Model 2: adjusted for age, sex, and residential area; and (3) Model 3 (fully-adjusted model): adjusted for all covariates in model 2 plus the CCI score. To test the robustness of our findings, we also performed subgroup analyses across the following of clinically relevant subgroups: age (65–69, 70–74, 75–80, and ≥ 80 years), sex (men and women), residential area (large city, small city, and rural area), and comorbidity burden (CCI scores: 0, 1, 2, and ≥ 3).

Next, to assess the associations between polypharmacy and the risk of hospitalization and death, we converted the number of medications into a binary exposure of polypharmacy (≥ 5 vs. 1–4 medications prescribed daily). This was then examined using the Cox models adjusted for age, sex, residential area, and CCI score in the overall and propensity-score matched cohorts. In establishing the propensity-score matched cohort, we matched individuals with and without polypharmacy (1:1 ratio) based on age, sex, residential area, and CCI scores. Further, time to the first occurrence of any hospitalization or death was also evaluated and compared between the two groups based on the number of medications or presence of polypharmacy using the Kaplan–Meier method and the log-rank test. There were no missing data in our study. All analyses were conducted using Stata version 15.1 (Stata Corporation, College Station, TX).

### Ethical approval

The current study was approved by the Institutional Review Board of the National Health Insurance Service Ilsan Hospital (NHIMC 2019-06-014).

### Informed consent

The Institutional Review Board of the National Health Insurance Service Ilsan Hospital waived the need for informed consent as only deidentified data were examined.

## Supplementary information

Supplementary Information.

## Data Availability

All relevant data are included in the manuscript and its supporting information files. Technical appendix and statistical code are available from Dr. Chang (email: kidneyjang@gmail.com).
