# Supplementary material for: Polypharmacy, hospitalization, and mortality risk: a nationwide cohort study
Source: Sci Rep. 2020 Nov 3;10:18964. doi: 10.1038/s41598-020-75888-8 (PMC7609640; doi:10.1038/s41598-020-75888-8)
Supplement: Supplementary file 1 — Supplementary Information. [file 41598_2020_75888_MOESM1_ESM.docx]

**Supplementary Information**

**Polypharmacy, Hospitalization, and Mortality Risk**

**: A Nationwide Cohort Study**

Authors:

Tae Ik Chang, MD, PhD; Haeyong Park, MSc; Dong Wook Kim, PhD;

Eun Kyung Jeon MSc; Connie M. Rhee MD, MSc;

Kamyar Kalantar-Zadeh, MD, MPH, PhD; Ea Wha Kang, MD, PhD;

Shin-Wook Kang, MD, PhD; and Seung Hyeok Han, MD, PhD

**Supplemental Tables**

**Supplementary Table S1.** Associations of the number of daily prescribed medications with adverse outcomes across various subgroups.

| Subgroup |  |  | Hospitalization |  |  |  | Death |  |
| --- | --- | --- | --- | --- | --- | --- | --- | --- |
|  |  | HR | 95% CI | *p* |  | HR | 95% CI | *p* |
| **Age, 65-69 years** |  |  |  |  |  |  |  |  |
| 1 to 2 |  | 1.00 |  |  |  | 1.00 |  |  |
| 3 to 4 |  | 1.05 | (1.05-1.06) | <0.001 |  | 1.12 | (1.09-1.14) | <0.001 |
| 5 to 6 |  | 1.15 | (1.14-1.16) | <0.001 |  | 1.27 | (1.23-1.30) | <0.001 |
| 7 to 8 |  | 1.26 | (1.24-1.27) | <0.001 |  | 1.43 | (1.39-1.47) | <0.001 |
| 8 to 10 |  | 1.37 | (1.35-1.39) | <0.001 |  | 1.61 | (1.55-1.67) | <0.001 |
| ≥11 |  | 1.55 | (1.53-1.57) | <0.001 |  | 1.84 | (1.77-1.90) | <0.001 |
| **Age, 70-74 years** |  |  |  |  |  |  |  |  |
| 1 to 2 |  | 1.00 |  |  |  | 1.00 |  |  |
| 3 to 4 |  | 1.05 | (1.04-1.06) | <0.001 |  | 1.08 | (1.06-1.10) | <0.001 |
| 5 to 6 |  | 1.13 | (1.12-1.14) | <0.001 |  | 1.24 | (1.22-1.27) | <0.001 |
| 7 to 8 |  | 1.23 | (1.21-1.24) | <0.001 |  | 1.40 | (1.37-1.43) | <0.001 |
| 8 to 10 |  | 1.32 | (1.31-1.33) | <0.001 |  | 1.57 | (1.53-1.61) | <0.001 |
| ≥11 |  | 1.46 | (1.44-1.47) | <0.001 |  | 1.75 | (1.71-1.80) | <0.001 |
| **Age, 75-79 years** |  |  |  |  |  |  |  |  |
| 1 to 2 |  | 1.00 |  |  |  | 1.00 |  |  |
| 3 to 4 |  | 1.03 | (1.02-1.04) | <0.001 |  | 1.09 | (1.07-1.11) | <0.001 |
| 5 to 6 |  | 1.10 | (1.09-1.11) | <0.001 |  | 1.23 | (1.20-1.25) | <0.001 |
| 7 to 8 |  | 1.19 | (1.18-1.20) | <0.001 |  | 1.36 | (1.33-1.39) | <0.001 |
| 8 to 10 |  | 1.27 | (1.26-1.29) | <0.001 |  | 1.46 | (1.43-1.50) | <0.001 |
| ≥11 |  | 1.40 | (1.38-1.41) | <0.001 |  | 1.61 | (1.57-1.65) | <0.001 |
| **Age, ≥80 years** |  |  |  |  |  |  |  |  |
| 1 to 2 |  | 1.00 |  |  |  | 1.00 |  |  |
| 3 to 4 |  | 1.03 | (1.02-1.04) | <0.001 |  | 1.05 | (1.04-1.06) | <0.001 |
| 5 to 6 |  | 1.09 | (1.08-1.11) | <0.001 |  | 1.13 | (1.11-1.14) | <0.001 |
| 7 to 8 |  | 1.17 | (1.16-1.18) | <0.001 |  | 1.19 | (1.17-1.21) | <0.001 |
| 8 to 10 |  | 1.24 | (1.22-1.25) | <0.001 |  | 1.23 | (1.21-1.26) | <0.001 |
| ≥11 |  | 1.32 | (1.30-1.34) | <0.001 |  | 1.28 | (1.25-1.30) | <0.001 |
| **Men** |  |  |  |  |  |  |  |  |
| 1 to 2 |  | 1.00 |  |  |  | 1.00 |  |  |
| 3 to 4 |  | 1.04 | (1.03-1.04) | <0.001 |  | 1.07 | (1.06-1.09) | <0.001 |
| 5 to 6 |  | 1.12 | (1.11-1.12) | <0.001 |  | 1.19 | (1.17-1.20) | <0.001 |
| 7 to 8 |  | 1.20 | (1.19-1.21) | <0.001 |  | 1.29 | (1.27-1.31) | <0.001 |
| 8 to 10 |  | 1.29 | (1.28-1.31) | <0.001 |  | 1.38 | (1.36-1.41) | <0.001 |
| ≥11 |  | 1.44 | (1.42-1.45) | <0.001 |  | 1.50 | (1.48-1.53) | <0.001 |
| **Women** |  |  |  |  |  |  |  |  |
| 1 to 2 |  | 1.00 |  |  |  | 1.00 |  |  |
| 3 to 4 |  | 1.05 | (1.05-1.06) | <0.001 |  | 1.09 | (1.07-1.10) | <0.001 |
| 5 to 6 |  | 1.14 | (1.13-1.14) | <0.001 |  | 1.21 | (1.20-1.23) | <0.001 |
| 7 to 8 |  | 1.24 | (1.23-1.24) | <0.001 |  | 1.34 | (1.32-1.36) | <0.001 |
| 8 to 10 |  | 1.33 | (1.32-1.34) | <0.001 |  | 1.44 | (1.42-1.47) | <0.001 |
| ≥11 |  | 1.46 | (1.45-1.47) | <0.001 |  | 1.61 | (1.58-1.63) | <0.001 |
| **Residential area: large city** |  |  |  |  |  |  |  |  |
| 1 to 2 |  | 1.00 |  |  |  | 1.00 |  |  |
| 3 to 4 |  | 1.06 | (1.05-1.06) | <0.001 |  | 1.09 | (1.07-1.10) | <0.001 |
| 5 to 6 |  | 1.15 | (1.14-1.16) | <0.001 |  | 1.20 | (1.18-1.22) | <0.001 |
| 7 to 8 |  | 1.26 | (1.25-1.27) | <0.001 |  | 1.33 | (1.31-1.36) | <0.001 |
| 8 to 10 |  | 1.36 | (1.35-1.37) | <0.001 |  | 1.43 | (1.41-1.46) | <0.001 |
| ≥11 |  | 1.52 | (1.50-1.53) | <0.001 |  | 1.59 | (1.56-1.62) | <0.001 |
| **Residential area: small city** |  |  |  |  |  |  |  |  |
| 1 to 2 |  | 1.00 |  |  |  | 1.00 |  |  |
| 3 to 4 |  | 1.05 | (1.04-1.05) | <0.001 |  | 1.08 | (1.06-1.09) | <0.001 |
| 5 to 6 |  | 1.12 | (1.11-1.13) | <0.001 |  | 1.20 | (1.18-1.22) | <0.001 |
| 7 to 8 |  | 1.21 | (1.20-1.22) | <0.001 |  | 1.30 | (1.28-1.32) | <0.001 |
| 8 to 10 |  | 1.31 | (1.29-1.32) | <0.001 |  | 1.40 | (1.37-1.42) | <0.001 |
| ≥11 |  | 1.43 | (1.41-1.44) | <0.001 |  | 1.52 | (1.49-1.55) | <0.001 |
| **Residential area: rural area** |  |  |  |  |  |  |  |  |
| 1 to 2 |  | 1.00 |  |  |  | 1.00 |  |  |
| 3 to 4 |  | 1.02 | (1.01-1.03) | <0.001 |  | 1.08 | (1.05-1.10) | <0.001 |
| 5 to 6 |  | 1.09 | (1.07-1.10) | <0.001 |  | 1.20 | (1.18-1.23) | <0.001 |
| 7 to 8 |  | 1.16 | (1.15-1.18) | <0.001 |  | 1.29 | (1.26-1.32) | <0.001 |
| 8 to 10 |  | 1.22 | (1.20-1.24) | <0.001 |  | 1.36 | (1.32-1.40) | <0.001 |
| ≥11 |  | 1.34 | (1.32-1.36) | <0.001 |  | 1.48 | (1.44-1.53) | <0.001 |
| **CCI score =0** |  |  |  |  |  |  |  |  |
| 1 to 2 |  | 1.00 |  |  |  | 1.00 |  |  |
| 3 to 4 |  | 1.06 | (1.06-1.07) | <0.001 |  | 1.08 | (1.06-1.10) | <0.001 |
| 5 to 6 |  | 1.20 | (1.19-1.21) | <0.001 |  | 1.20 | (1.17-1.23) | <0.001 |
| 7 to 8 |  | 1.34 | (1.32-1.36) | <0.001 |  | 1.30 | (1.26-1.34) | <0.001 |
| 8 to 10 |  | 1.48 | (1.44-1.51) | <0.001 |  | 1.42 | (1.35-1.49) | <0.001 |
| ≥11 |  | 1.66 | (1.61-1.72) | <0.001 |  | 1.64 | (1.54-1.75) | <0.001 |
| **CCI score =1** |  |  |  |  |  |  |  |  |
| 1 to 2 |  | 1.00 |  |  |  | 1.00 |  |  |
| 3 to 4 |  | 1.03 | (1.03-1.04) | <0.001 |  | 1.10 | (1.08-1.12) | <0.001 |
| 5 to 6 |  | 1.12 | (1.11-1.13) | <0.001 |  | 1.23 | (1.20-1.25) | <0.001 |
| 7 to 8 |  | 1.22 | (1.21-1.23) | <0.001 |  | 1.35 | (1.32-1.38) | <0.001 |
| 8 to 10 |  | 1.34 | (1.32-1.36) | <0.001 |  | 1.48 | (1.44-1.52) | <0.001 |
| ≥11 |  | 1.47 | (1.44-1.49) | <0.001 |  | 1.62 | (1.57-1.67) | <0.001 |
| **CCI score =2** |  |  |  |  |  |  |  |  |
| 1 to 2 |  | 1.00 |  |  |  | 1.00 |  |  |
| 3 to 4 |  | 1.02 | (1.02-1.03) | <0.001 |  | 1.09 | (1.07-1.11) | <0.001 |
| 5 to 6 |  | 1.09 | (1.08-1.10) | <0.001 |  | 1.25 | (1.23-1.28) | <0.001 |
| 7 to 8 |  | 1.19 | (1.18-1.20) | <0.001 |  | 1.38 | (1.35-1.41) | <0.001 |
| 8 to 10 |  | 1.28 | (1.26-1.29) | <0.001 |  | 1.46 | (1.42-1.50) | <0.001 |
| ≥11 |  | 1.41 | (1.40-1.43) | <0.001 |  | 1.63 | (1.59-1.67) | <0.001 |
| **CCI score ≥3** |  |  |  |  |  |  |  |  |
| 1 to 2 |  | 1.00 |  |  |  | 1.00 |  |  |
| 3 to 4 |  | 1.02 | (1.02-1.03) | <0.001 |  | 1.03 | (1.01-1.05) | 0.001 |
| 5 to 6 |  | 1.07 | (1.06-1.08) | <0.001 |  | 1.11 | (1.09-1.13) | <0.001 |
| 7 to 8 |  | 1.15 | (1.14-1.16) | <0.001 |  | 1.22 | (1.20-1.24) | <0.001 |
| 8 to 10 |  | 1.24 | (1.22-1.25) | <0.001 |  | 1.31 | (1.29-1.34) | <0.001 |
| ≥11 |  | 1.38 | (1.37-1.39) | <0.001 |  | 1.44 | (1.41-1.46) | <0.001 |

*Note:* All models were adjusted for age, sex, residential area, and Charlson comorbidity index score. *Abbreviations:* HR, hazard ratio; CI, confidence interval; CCI, Charlson comorbidity index.

**Supplementary Table S2.** Selected individual characteristics stratified by polypharmacy status in each cohort.

|  |  | Overall cohort | | |  | Propensity score-matched cohort | | |
| --- | --- | --- | --- | --- | --- | --- | --- | --- |
|  |  |  | Polypharmacy | |  |  | Polypharmacy | |
| Characteristic |  | Overall | Absence | Presence |  | Overall | Absence | Presence |
| Number of participants |  | 3,007,620 | 1,606,171 | 1,401,449 |  | 2,140,674 | 1,070,337 | 1,070,337 |
| Number of medications |  | 4.9±3.2 | 2.6±1.1 | 7.6±2.7 |  | 5.0±3.1 | 2.6±1.1 | 7.4±2.6 |
| Age, years |  | 73.4±6.3 | 72.9±6.3 | 73.9±6.2 |  | 73.5±6.3 | 73.5±6.2 | 73.5±6.3 |
| 65-69 years, % |  | 31.5 | 35.2 | 27.3 |  | 30.2 | 30.2 | 30.2 |
| 70-74 years, % |  | 31.0 | 31.0 | 31.0 |  | 31.6 | 31.6 | 31.6 |
| 75-79 years, % |  | 20.7 | 18.9 | 22.8 |  | 21.1 | 21.1 | 21.1 |
| ≥80 years, % |  | 16.9 | 15.0 | 19.0 |  | 17.1 | 17.1 | 17.1 |
| Gender, % men |  | 39.5 | 39.4 | 39.7 |  | 39.6 | 39.6 | 39.6 |
| Residential area, % |  |  |  |  |  |  |  |  |
| Large city |  | 43.5 | 44.2 | 42.8 |  | 42.8 | 42.8 | 42.8 |
| Small city |  | 42.8 | 42.5 | 43.2 |  | 42.8 | 42.8 | 42.8 |
| Rural area |  | 13.5 | 13.3 | 14.1 |  | 14.4 | 14.4 | 14.4 |
| CCI scores |  | 2.0±1.7 | 1.5±1.5 | 2.6±1.8 |  | 2.1±1.6 | 2.0±1.5 | 2.1±1.7 |
| 0, % |  | 18.5 | 27.2 | 8.6 |  | 11.3 | 11.3 | 11.3 |
| 1, % |  | 27.6 | 31.9 | 22.7 |  | 29.7 | 29.7 | 29.7 |
| 2, % |  | 22.2 | 20.8 | 23.8 |  | 28.7 | 28.7 | 28.7 |
| ≥3, % |  | 31.7 | 20.2 | 44.9 |  | 30.3 | 30.3 | 30.3 |

*Note:* Data are presented as means ± standard deviation, number, or percentages. *Abbreviations:* CCI, Charlson comorbidity index.

**Supplementary Table S3.** Fully adjusted Cox models examining the association between polypharmacy and adverse outcomes in the overall and propensity-score matched cohorts.

| Outcome |  | Overall cohort | | |  | Propensity score-matched cohort | | |
| --- | --- | --- | --- | --- | --- | --- | --- | --- |
|  |  | HR | 95% CI | *p* |  | HR | 95% CI | *p* |
| **Hospitalization** |  |  |  |  |  |  |  |  |
| Presence of polypharmacy (vs absence) |  | 1.18 | (1.18-1.19) | <0.001 |  | 1.16 | (1.16-1.17) | <0.001 |
| Age (per 1 year increase) |  | 1.02 | (1.02-1.02) | <0.001 |  | 1.02 | (1.02-1.02) | <0.001 |
| Men (vs women) |  | 1.00 | (1.00-1.00) | 0.958 |  | 1.00 | (1.00-1.00) | 0.421 |
| Small city (vs large city) |  | 1.07 | (1.07-1.08) | <0.001 |  | 1.08 | (1.07-1.08) | <0.001 |
| Rural area (vs large city) |  | 1.24 | (1.23-1.25) | <0.001 |  | 1.24 | (1.23-1.24) | <0.001 |
| CCI score (per 1 point increase) |  | 1.09 | (1.09-1.09) | <0.001 |  | 1.08 | (1.08-1.08) | <0.001 |
| **Death** |  |  |  |  |  |  |  |  |
| Presence of polypharmacy (vs absence) |  | 1.25 | (1.24-1.25) | <0.001 |  | 1.25 | (1.24-1.25) | <0.001 |
| Age (per 1 year increase) |  | 1.12 | (1.12-1.12) | <0.001 |  | 1.12 | (1.12-1.12) | <0.001 |
| Men (vs women) |  | 1.40 | (1.39-1.41) | <0.001 |  | 1.41 | (1.40-1.42) | <0.001 |
| Small city (vs large city) |  | 1.04 | (1.03-1.04) | <0.001 |  | 1.04 | (1.03-1.05) | <0.001 |
| Rural area (vs large city) |  | 1.11 | (1.10-1.12) | <0.001 |  | 1.11 | (1.10-1.12) | <0.001 |
| CCI score (per 1 point increase) |  | 1.10 | (1.10-1.11) | <0.001 |  | 1.11 | (1.11-1.11) | <0.001 |

*Note:* All models were adjusted for age, sex, residential area, and Charlson comorbidity index score. *Abbreviations:* HR, hazard ratio; CI, confidence interval; CCI, Charlson comorbidity index.

**Supplemental Materials**

**Supplemental Figures**


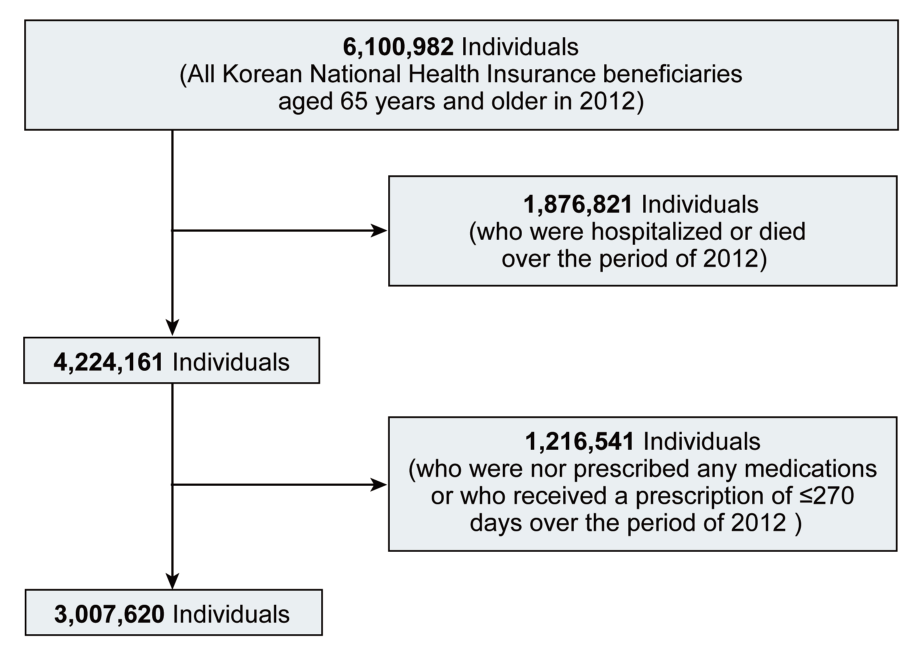


**Supplementary Figure S1.** Flow chart of study cohort construction. *Abbreviations:* NHIS, National Health Insurance Service; eGFR, estimated glomerular filtration rate.





**Supplementary Figure S2.** The Kaplan-Meier plots for (A) hospitalization and (B) mortality in the overall cohort according to the number of prescription medications.





**Supplementary Figure S3.** The Kaplan-Meier plots for (A) hospitalization and (B) mortality in the overall cohort according to presence of polypharmacy.





**Supplementary Figure S4.** The Kaplan-Meier plots for (A) hospitalization and (B) mortality in the propensity-score matched cohort according to presence of polypharmacy.
